# High Levels of Circulating IL-8 and Soluble IL-2R Are Associated With Prolonged Illness in Patients With Severe COVID-19

**DOI:** 10.3389/fimmu.2021.626235

**Published:** 2021-01-29

**Authors:** Aiping Ma, Liang Zhang, Xiaokai Ye, Jing Chen, Jie Yu, Liangjin Zhuang, Chaohang Weng, Frank Petersen, Zhanxiang Wang, Xinhua Yu

**Affiliations:** ^1^ Department of Respiratory and Critical Medicine, The First Affiliated Hospital, School of Medicine, Xiamen University, Xiamen, China; ^2^ Priority Area Asthma & Allergy, Research Center Borstel, Airway Research Center North (ARCN), Members of the German Center for Lung Research (DZL), Borstel, Germany; ^3^ Department of Respiratory and Critical Medicine, Xiamen Haicang Hospital, Xiamen, China; ^4^ Department of Critical Care Medicine, The First Affiliated Hospital, School of Medicine, Xiamen University, Xiamen, China; ^5^ Department of Anesthesiology, Tongji Hospital, Tongji Medical College, Huazhong University of Science and Technology, Wuhan, China; ^6^ Division of Quality Management, The First Affiliated Hospital, School of Medicine, Xiamen University, Xiamen, China; ^7^ Department of Neurosurgery, The First Affiliated Hospital, School of Medicine, Xiamen University, Xiamen, China

**Keywords:** soluble interleukin 2 receptor, interleukin 8, interleukin 6, neutrophil-to-lymphocytes ratio, severe acute respiratory syndrome coronavirus−2, coronavirus disease 2019

## Abstract

**Objectives:**

The coordinated immune response of the host is the key of the successful combat of the body against SARS-CoV-2 infection and is decisive for the development and progression of COVID-19. In this study, we aimed to investigate whether the immunological phenotype of patients are associated with duration of illness in patients with severe COVID-19.

**Method:**

In this single-center study, 69 patients with severe or critical COVID-19 were recruited retrospectively. Immunological parameters including counts of white blood cells, neutrophils, lymphocytes, the neutrophil-to-lymphocyte ratio, and levels of circulating cytokines and cytokine receptors were screened for their association with disease severity, survival and duration of illness of COVID-19.

**Results:**

Our data confirmed previous results that neutrophil-to-lymphocyte ratio and circulating levels of IL-6 represent prominent biomarker for the prediction of disease severity and survival of COVID-19. However, this study shows for the first time that duration of illness in patients with severe COVID-19 is positively associated with serum levels of IL-8 (*P*=0.004) and soluble IL-2Rα (*P*=0.025).

**Conclusion:**

The significant association of duration of illness with circulating levels of IL-8 and soluble IL-2Rα in patients with severe COVID-19 implicates that neutrophils and T cells are involved in the evolution of COVID-19.

## Introduction

Coronavirus disease 2019 (COVID-19) which was first reported in Wuhan, China is viral pneumonia caused by severe acute respiratory syndrome coronavirus 2 (SARS-CoV-2) (1, 2). On 11 March 2020, the World Health Organization (WHO) declared COVID-19 as a pandemic (3). As of Dec. 20, 2020, over 77 million cases of COVID19 worldwide have been laboratory-confirmed, with more than 1.7 million deaths (4). According to the WHO guideline, approximately 80% of patients with COVID-19 may have mild to moderate disease which needs no or limited inpatient care; while the test 20% of patients have severe or critical illness disease and require intensive inpatient interventions (5). In general, elderly people and patients with comorbidities are more susceptible to the severe illness and death (6, 7), the case-fatality-ratio varies considerably among populations (8).

The entry of SARS-CoV-2 is dependent on the surface-expressed angiotensin-converting enzyme 2 (ACE2) on human host cells (9). SARS-CoV-2 infection activates both innate and adaptive immune responses in the host (10, 11). Optimal and well-coordinated immune responses are able to eliminate the virus without causing tissue injury and thus lead to asymptomatic or mild forms of disease. By contrast, excessive and dysregulated immune responses can result in inordinate inflammation and harmful tissue damage at both local and systemic levels, causing severe illness (10, 11). Within this view, some immunological parameters have been identified as prominent biomarker to predict the severity of the disease and patient survival rates, including neutrophil-to-lymphocytes ratio (NLR) in peripheral blood and circulating levels of interleukin-6 (IL-6) (12–15). Duration of illness, the time-interval between the onset and the clinical recovery, is an important parameter of the disease progression and recovery. In this study, we investigated whether immunological parameters including immune cells in peripheral blood and circulating cytokines are associated with duration of illness in patients with severe COVID-19.

## Materials and Methods

### Patients and Data Collection

We retrospectively recruited 69 patients with severe or critical COVID-19 from February 10 to March 28, 2020, at Guanggu Branch of Tongji Hospital, Huazhong University of Science and Technology in Wuhan (China). The hospital was urgently reconstructed and assigned as a designated hospital for admitting patients with severe COVID-19. All patients with COVID-19 were diagnosed and classified according to the diagnosis and treatment protocols of COVID-19 (6th edition) released by the National Health Commission of China (16). A patient was diagnosed as a severe case when any of the following criteria was met: 1) respiratory distress with the respiratory rate over 30 per minute, 2) oxygen saturation ≤ 93% in the resting state, and 3) arterial blood oxygen partial pressure (PaO2)/oxygen concentration (FiO2) ≤300mmHg. A critically ill case was diagnosed when any of the following criteria was met: 1) respiratory failure occurs and requires mechanical ventilation; 2) shock; 3) other organ failure requiring intensive care were classified as critical illness. A *patient was* considered “ready for *discharge*” when all four *criteria were met* concurrently, including 1) afebrile for greater than 3 days; 2) respiratory symptoms significantly improved; 3) improvement in the radiological abnormalities on chest radiograph or CT; and 4) two consecutive negative COVID-19 nucleic acid tests at least 24 h apart (16). The duration of illness was defined as time from onset of disease to hospital discharge. This study was performed in accordance with the 1964 Helsinki Declaration and its later amendments or comparable ethical standards, and the approval was obtained from the Institutional Review Board of Tongji Hospital, Huazhong University of Science and Technology. Since this retrospective study contains solely anonymized data, informed consent was waived by the Institutional Review Board.

We obtained demographic, clinical, treatment, and outcome data from electronic medical records. Before proceeding to any analysis, data were fully anonymized by removing personally *identifiable* information. Hematological parameters were determined for all 69 patients within the first 2 days of hospital admission using SYSMEX XT-1800i automated hematology analyzers (Sysmex, Kobe, Japan). Since determination of cytokines was performed as a routine test for COVID-19 patients only after the middle of February, only 54 patients were tested for serum levels of cytokines and cytokine receptors including IL-1β, soluble IL-2 receptor alpha (sIL-2Rα), IL-6, IL-8, IL-10, and TNF-α using chemiluminescence immunoassay (CLIA) performed on a fully automated analyzer (Siemens IMMULITE 1000, DiaSorin LIAISON, or Roche Diagnostics Cobas e602) according to the manufacturers’ instructions. The data were reviewed independently by two physicians of the Wuhan-Xiamen Medical Treatment Group for COVID-19.

### Statistical Analysis

All statistical calculations were performed using R software (version 4.0.2). Continuous variables were expressed as the appropriate means (range) and standard deviations. Categorical variables were summarized as the counts (percentages) in each category (17). Optimal cut-off values of the continuous variables for the prediction of severity, survival, and duration of illness were determined by applying the receiver operating curve (ROC) analysis (17). To generate the ROC curves, patients were categorized into two groups according to disease severity (severe illness, critical illness), survival (survival, death), and duration of illness (more than or equal to average, less than average). The optimal cut-off values were determined by minimizing the Manhattan distance on the ROC curve to the left top edge of the diagram where the sum of sensitivity and specificity was maximized. With optimal outpoints, laboratory variables were converted from continuous to binary and used for determining their association with clinical outcomes. To determined difference between groups, Wilcoxon rank sum test was applied to continuous variables, and Fisher exact test was used for categorical variables (17, 18). Correlations between variables were analyzed by the “rcorr” function in R, and P values and correlation coefficient were calculated. *P* values for difference between two groups of curves were calculated by the Log rank test of the “ggsurvplot” function. *P* values obtained from multiple testing were adjusted using the Bonferroni correction to avoid false positives, and an analysis with *P*< 0.05 was considered statistically significant.

## Results

### Demographic, Clinical, and Laboratory Characteristics

Among the 69 patients (41 females and 28 males) with COVID-19 recruited in this study, 63 and 6 had developed severe illness and critical illness, respectively. The demographic and clinical characteristics are summarized in **Table 1**. The median age of patients was 58 years, ranging from 30 to 86 years. The most common initial symptom was fever (51/69, 73.9%), followed by cough (34/69, 49.3%). The most common comorbidity was hypertension (13/68, 19.1%), followed by diabetes (3/68, 4.4%). The mean time interval from the onset of disease to admission was 18.7 days, and the average hospitalization time was 19.8 days. All patients received antiviral therapy and the majority of patients (85.1%) were also treated with antibiotics. Regarding the clinical outcomes, three patients died, one was transferred to another hospital and could not be followed, and remaining 65 patients were discharged after recovery, with an average time interval from onset to clinical recovery of 38.4 days. As compared to patients with severe illness, patients with critical illness showed higher mortality rate (40 *vs*. 1.59%, *P*=0.013), but this difference was not significant after correction for multiple comparisons.

**Table 1:** Demographic and clinical features of patients with COVID-19.

	Total (n = 69)	Severe illness (n = 63)	Critical illness (n = 6)	*P* value	Adjusted *P* value
**Age**	58 (30–86)	59 (30–81)	69 (44–86)	Ns	Ns
**Gender**				Ns	Ns
Male	28 (40.58%)	24 (38.10%)	4 (66.67%)		
Female	41 (59.42%)	39 (61.90%)	2 (33.33%)		
**Initial symptom**					
Fever	30 (43.48%)	29 (46.03%)	1 (16.67%)	Ns	Ns
Cough	12 (17.39%)	10 (15.87%)	2 (33.33%)	Ns	Ns
Fever and cough	17 (24.64%)	15 (23.81%)	2 (33.33%)	Ns	Ns
Dyspnea	1 (1.45%)	0 (0%)	1 (16.67%)	Ns	Ns
Cough and dyspnea	1 (1.45%)	1 (1.59%)	0 (0%)	Ns	Ns
Fever, cough, and dyspnea	4 (5.80%)	4 (6.35%)	0 (0%)	Ns	Ns
Chest tightness	2 (2.90%)	2 (3.17%)	0 (0%)	Ns	Ns
Breathlessness	1 (1.45%)	1 (1.59%)	0 (0%)	Ns	Ns
No-symptom	1 (1.45%)	1 (1.59%)	0 (0%)	Ns	Ns
Comorbidity*					
Hypertension	13 (19.12%)	10 (16.13%)	3 (50%)	Ns	Ns
Diabetes	3 (4.41%)	2 (3.23%)	1 (16.67%)	Ns	Ns
Coronary heart disease	1 (1.47%)	0 (0%)	1 (16.67%)	Ns	Ns
Stroke	1 (1.47%)	1 (1.61%)	0 (0%)	Ns	Ns
Gout	1 (1.47%)	1 (1.61%)	0 (0%)	Ns	Ns
Treatment**					
Antiviral therapy	67 (100%)	61 (100%)	6 (100%)	Ns	Ns
Antibiotic	57 (85.10%)	53 (86.89%)	4 (66.7%)	Ns	Ns
Clinical parameters***					
Discharged	65 (95.6%)	62 (98.41%)	3 (60%)	0.013	Ns
Died	3 (4.41%)	1 (1.59%)	2 (40%)	0.013	Ns
Days from onset to discharge	38.42 (8–82)	38.90 (10–82)	33.33 (8–61)	Ns	Ns
Days from onset to admission	18.65 (0–54)	18.78 (0–54)	17.33 (0–49)	Ns	Ns
Day from onset to cytokine test	26.52 (0–54)	26.83 (0–54)	22.50 (0–49)	Ns	Ns

Age and days are represented as mean (range), P values were calculated by Wilcoxon rank sum tests. Categorical variables are represented as number (%), P value were calculated by Fisher exact test. P values were adjusted for multiple comparison using Bonferroni correction method. Ns, not significant. *Information of comorbidity of one patient was missing. **treatment information was available for 67 patients. ***Clinical outcome of one patient with critical illness is not clear since the patient was transferred to another hospital.

Laboratory data including hematological parameters as well as serum levels of cytokines or cytokine receptors of the patients were summarized in **Table 2**. Patients with critical illness showed significantly higher levels of white blood cells (WBC) (12.28 x 10^9^/ml *vs.* 5.9 x 10^9^/ml, *P*=0.014, *P^adjusted^*=0.042), neutrophils (9.95 x 10^9^/ml *vs.* 3.91 x 10^9^/ml, *P*=0.006, *P^adjusted^*=0.030), and neutrophil-to-lymphocytes ratio (NLR) (11.41 *vs.* 3.78, *P*=0.014, *P^adjusted^*=0.042) than patients with severe illness. Furthermore, serum levels of interleukin 6 (IL-6) (43.32 *vs.* 5.47 pg/ml, *P*=0.001, *P^adjusted^*=0.008), IL-10 (23.68 *vs.* 5.60 pg/ml, *P*=0.001, *P^adjusted^*=0.008), and soluble IL-2 receptor alpha (sIL-2Rα) (1,017 *vs.* 452 U/ml, *P*=0.031, *P^adjusted^*=0.078) were increased in patients with critical illness compared to those with a severe status.

**Table 2:** Laboratory characteristics of patients with COVID-19.

	Total	Severe illness	Critical illness	*P* value	Adjusted *P* value
WBC (x10^9^/ml)	6.47 (2.28–20.43)	5.91 (2.28–13.71)	12.28 (3.25–20.43)	0.014	0.042
Neutrophils (x10^9^/ml)	4.43 (1.27–18.73)	3.91 (1.27–12.62)	9.95 (2.42–18.73)	0.006	0.030
Lymphocytes (x10^9^/ml)	1.41 (0.35–5.47)	1.39 (0.40–3.80)	1.59 (0.35–5.47)	Ns	Ns
NLR	4.44 (0.8–26.29)	3.78 (0.80–26.29)	11.41 (1.45–21.20)	0.014	0.042
RBC (x10^12^/ml)	4.07 (2.65–6.61)	4.09 (2.65–6.61)	3.95 (2.69–5.01)	Ns	Ns
PLT (x10^9^/ml)	245.81 (52–659)	250.95 (52–659)	191.83 (103–361)	Ns	Ns
sIL-2Rα* (U/ml)	494.37 (51–1,468)	452.56 (51–1,468)	1,017 (370–1,440)	0.031	Ns
IL-6* (pg/ml)	8.21 (1.5–52.2)	5.47 (1.5–38.97)	43.32 (26.26–52.2)	0.001	0.008
IL-8* (pg/ml)	14.91 (5–189)	13.68 (5–189)	30.28 (7.6–85.2)	Ns	Ns
IL-10* (pg/ml)	6.94 (5–69.1)	5.60 (5–24.3)	23.68 (5–69.1)	0.001	0.008
TNF-α* (pg/ml)	8.14 (4–25.4)	7.65 (4–17.5)	14.18 (6.9–25.4)	Ns	Ns

All laboratory data were represented by mean (range), P values were calculated by Wilcoxon rank sum tests and adjusted for multiple comparison using Bonferroni correction method. Ns, not significant; WBC, white blood cells; RBC, red blood cells; NLR, neutrophil-to-lymphocyte ratio; PLT, platelets. *Only there laboratory characteristics were tested only in 54 patients.

### Association of Immunological Parameters With Clinical Outcomes

**Figure 1:**
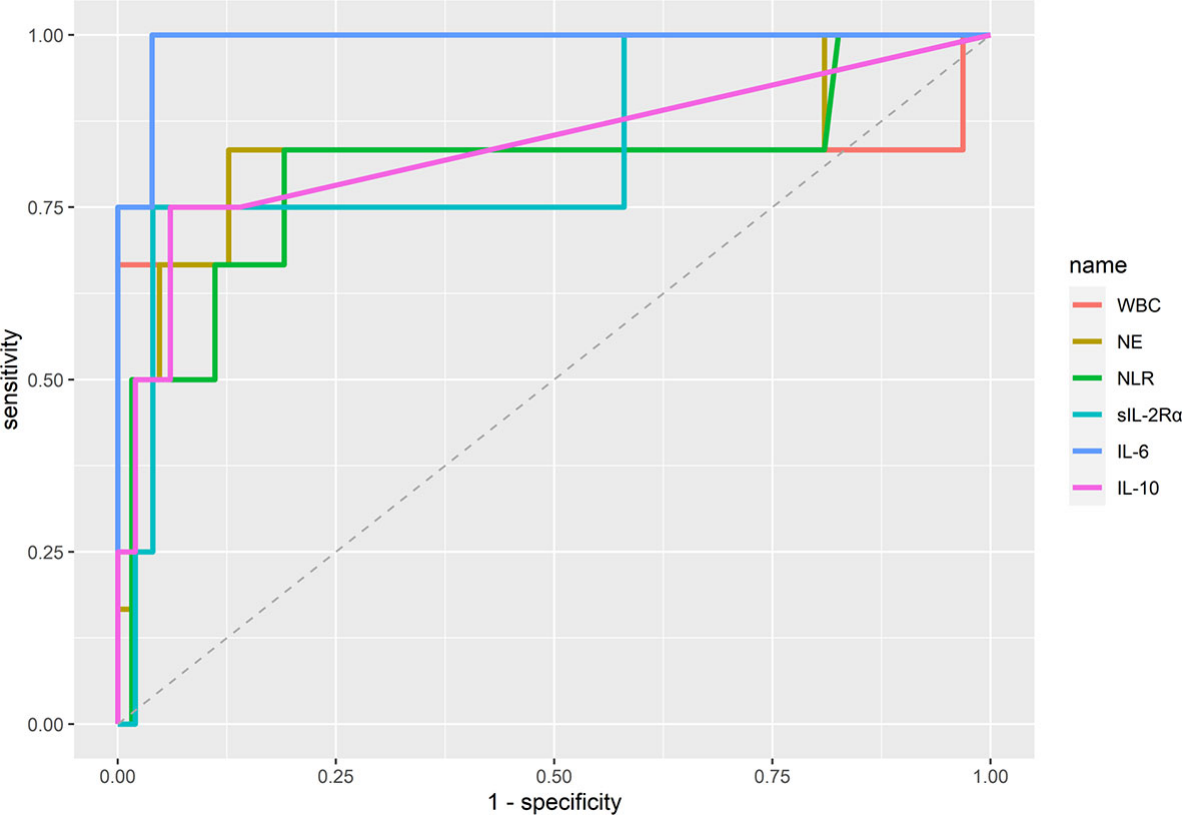
Diagnostic accuracy of immunological parameters distinguishing between COVID-19 patients with severe and critical illness. Area under curve (AUC) was calculated by using the “auc” function in R. The optimal cut-off values (threshold) of the diagnostic markers were calculated by applying the receiver operating curve (ROC) analysis. WBC, white blood cells; NLR, neutrophil-to-lymphocyte ratio; NE, neutrophils.

We first investigated whether specific laboratory parameters could be used as diagnostic biomarkers for distinguishing critical illness from severe illness. Receiver operating characteristic (ROC) curve analysis was performed to calculate the area under curve (AUC) of each ROC curve. As shown in **Figure 1** and **Supplementary Table 1**, the most efficient diagnostic mark to distinguish the two groups was the IL-6 concentration in sera [threshold=24.92 pg/ml, AUC=0.990 (0.967–1.000)], followed by IL-10 levels (threshold=6.85 pg/ml, AUC=0.837 (0.568–1.000)), neutrophil counts [threshold=5.79 x 10^9^/ml, AUC=0.831 (0.575–1.000)], sIL-2Rα concentrations [threshold=957 U/ml, AUC=0.83 (0.557–1.000)], WBC counts [threshold=13.75 x 10^9^/ml, AUC=0.807 (0.496–1.000)], and the NLR [threshold=4.94, AUC=0.806 (0.551–1.000)].

**Figure 2:**
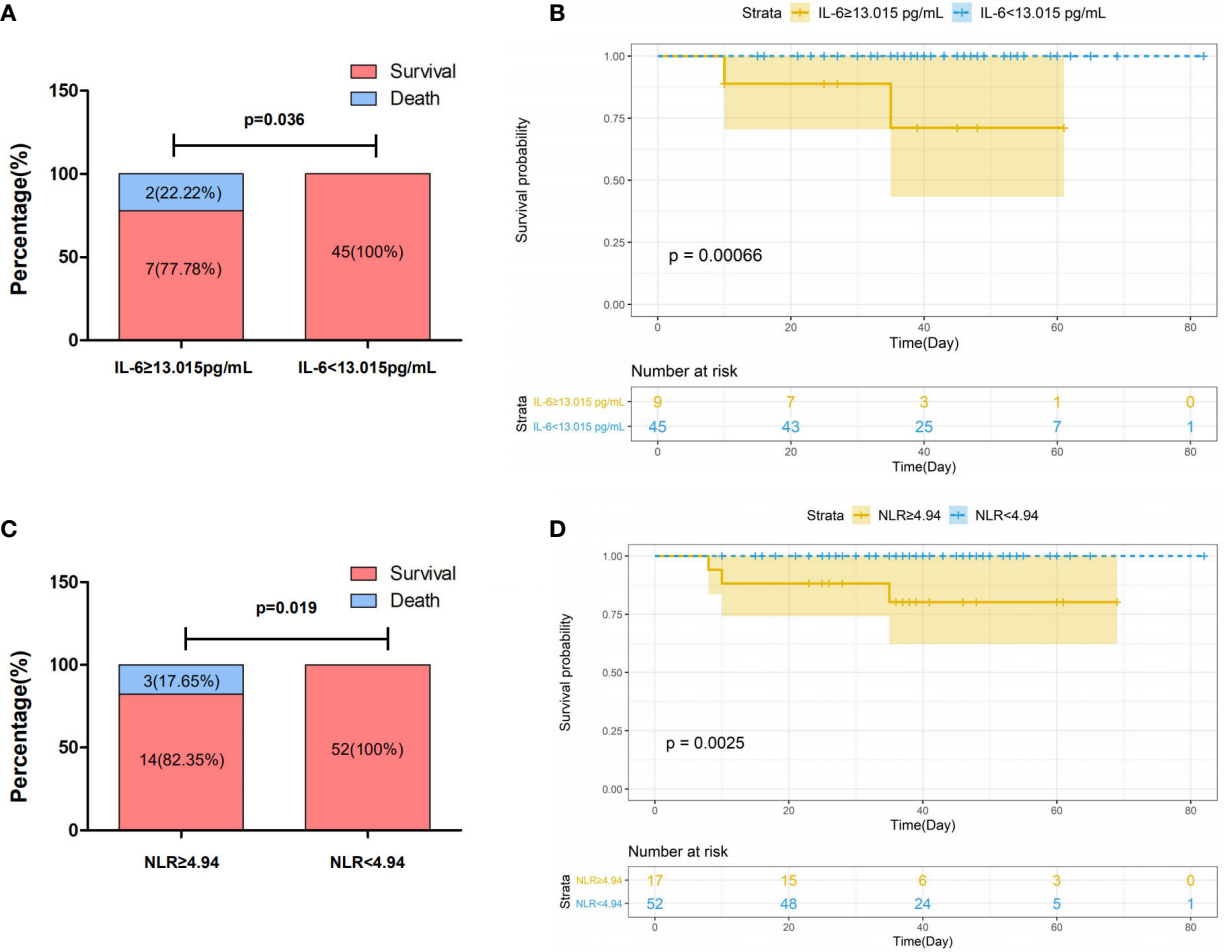
Association of survival with IL-6 **(A, B)** and NLR **(C, D)** in patients with severe COVID-19. Patients with COVID-19 were categorized into two group according to the threshold indicated in the figure. Survival rates in patients with COVID-19 stratified by levels of IL-6 **(A)** and NLR **(C)**. P values for difference between survival rates were calculated using Fisher exact test. Kaplan–Meier survival curves in patients with COVID-19 stratified by IL-6 **(B)** and NLR **(D)** with confidence interval were generated by R. *P* values for difference between two groups of curves were calculated by the log rank test of the “ggsurvplot”.

Next, we evaluated the association of laboratory data with survival in COVID-19. Laboratory data were converted from continuous to binary according to optimal cut-off values determined by applying ROC analysis (**Supplementary Figure 1**). As shown in **Figure 2A**, the survival rate was significantly associated with serum levels of IL-6, where patients with low levels (<13.015 pg/ml) showed an increased survival rate as compared to those with high values (≥13.015) (100 *vs*. 77.78%, *P*=0.036). A comparable association was seen between survival rate and NLR (100 *vs*. 82.35%, *P*=0.019) (**Figure 2C**). The association was confirmed by the survival analysis using Kaplan-Meier estimate, where IL-6 (*P*=0.00066) and NLR (*P*=0.0025) were shown as efficient prognostic biomarkers (**Figures 2B, D**).

**Figure 3:**
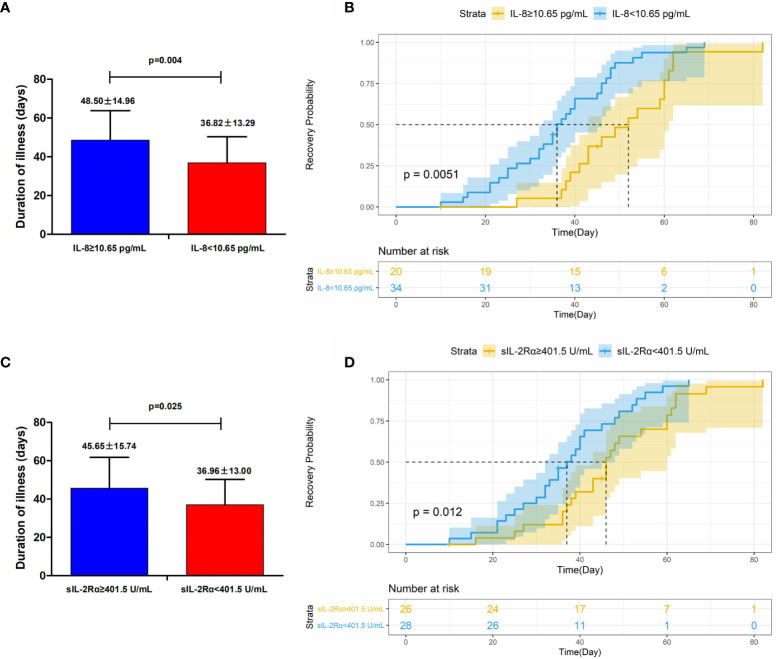
Association of duration of illness and IL-8 **(A, B)** and sIL-2Rα **(C, D)**. Patients with COVID-19 were categorized two group according to the threshold indicated in the figure. Duration of illness in patients with COVID-19 stratified by levels of IL-8 **(A)** and sIL-2Rα **(C)**. *P* values for difference in duration of illness were calculated using Wilcoxon rank sum test. Recovery curves in patients with COVID-19 stratified by IL-8 **(B)** and sIL-2Rα **(D)**. Recovery curves with confidence intervals were generated by R. P values for difference between two groups of curves were calculated by the Log rank test of the “ggsurvplot” function.

Then we analyzed the potential association between duration of illness of patients and different immunological parameters, including immune cells and cytokines. The most significant association was observed here between duration of illness and serum levels of IL-8. Patients with high levels of IL-8 (≥10.65 pg/ml) experienced a significantly longer duration of illness than those with low levels (<10.65 pg/ml) (48.5 ± 14.96 *vs.* 36.82 ± 13.29 days, *P*=0.004) (**Supplementary Figure 2A**, **Figure 3A**). In addition, a similar positive association was seen between duration of illness and serum levels of sIL-2Rα where high levels of sIL-2Rα (≥401.5 U/ml) were associated with longer duration of illness (45.65 ± 15.74 *vs.* 36.96 ± 13.00 days, *P*=0.025) (**Supplementary Figure 2B**, **Figure 3C**). Furthermore, analysis of the curve of recovery also confirmed the association of duration of illness with IL-8 and sIL-2Rα (**Figures 3B, D**).

**Figure 4:**
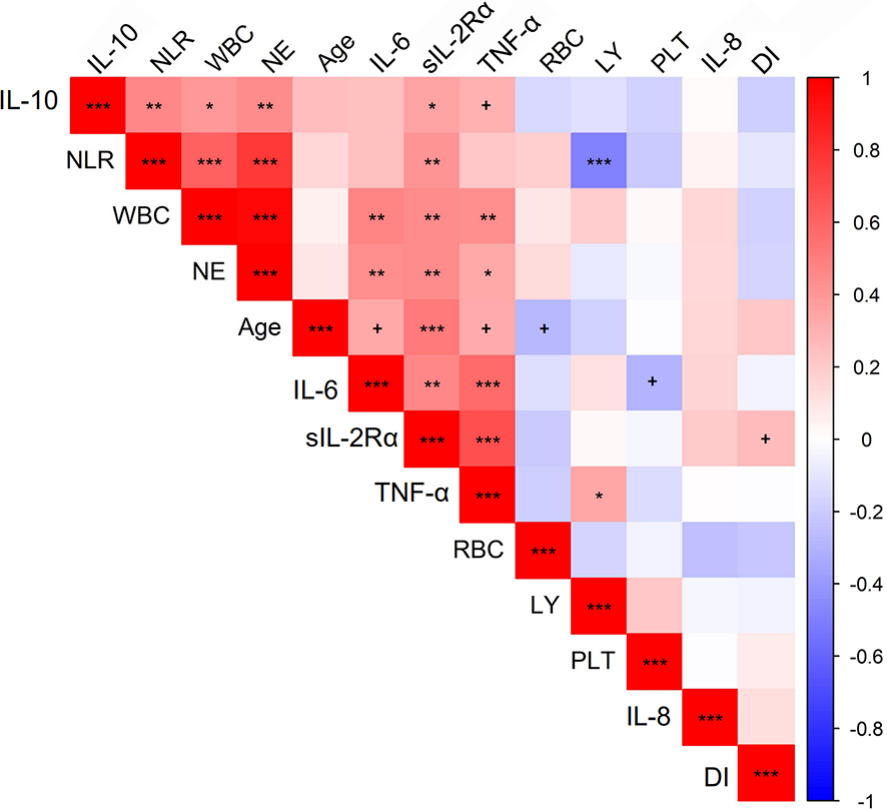
Correlations between quantitative traits in COVID-19 patients. Correlations between variables were analyzed by the “rcorr” function in R, and P values and correlation coefficient were calculated. The color of squares in the figure stands for correlation coefficient. P values were calculated and adjusted for multiple comparison. ^+^ *P* < 0.05 but *P^adjusted^ > *0.05, *P^adjusted^ < 0.05, **P^adjusted^ < 0.01, ***P^adjusted^ < 0.001. The picture was plotted by R package “corrplot.” NLR, neutrophil-to-lymphocyte ratio; WBC, white blood cell; NE, neutrophil count; LY, lymphocytes; RBC, red blood cells; PLT, platelets; DI, duration of illness.

Finally we analyzed potential correlations among paired continuous laboratory or clinical variables. As shown in **Figure 4** and **Supplementary Table 2**, significant correlations were identified several paired parameters. As expected, NLR was positively correlated with levels of WBC (r=0.62, *P^adjusted^*<0.001) and neutrophils (r=0.77, *P^adjusted^*<0.001) and negatively associated with levels of lymphocytes (r=-0.49, *P^adjusted^*<0.001). Significant correlations were also observed between circulating immune cells and inflammatory cytokines. For example, both circulating WBC and neutrophils were positively correlated with levels of TNF-α, IL-6, and IL-10. With regards to sIL-2Rα and IL-8 which was revealed to be associated with duration of illness of COVID-19 in this study, levels of sIL-2Rα were positively correlated with age (r=0.52, *P^adjusted^*<0.001), TNF-α (r=0.68, *P^adjusted^*<0.001), IL-6 (r=0.47, *P^adjusted^*<0.01), IL-10 (r=0.36, *P^adjusted^*<0.05), WBC (r=0.44, *P^adjusted^*<0.01), neutrophils (r=0.45, *P^adjusted^*<0.01), and NLR (r=0.41, *P^adjusted^*<0.01), while no significant correlation was revealed between IL-8 and any parameters. In addition, levels of the sIL-2Rα represent the only parameter which is marginally correlated with the duration of illness (r=0.27, *p*<0.05 but *P^adjusted^*>0.05).

## Discussion

In the current study, we evaluated the relationship between immune response-associated laboratory variables and clinical features in patients with severe COVID-19. Our analysis demonstrated that several immunological parameters are associated with severity, survival, and the duration of illness in patients with severe COVID-19.

Laboratory data have been intensively analyzed in the past for searching diagnostic and prognostic biomarkers to predict the severity and survival of COVID-19. Among hematological parameters, NLR has been convincingly shown as efficient biomarker to predict the severity and survival of the disease (14, 15, 18, 19), which is based on the phenomenon that patients with COVID-19 develop neutrophilia in combination with lymphopenia (1, 20). Among circulating cytokines, IL-6 has been demonstrated in several studies to be associated with severity and survival in patients with COVID-19 (12, 13, 21). Our study confirms these findings and shows that both NLR and levels of IL-6 were associated with severity and survival of COVID-19. Intriguingly, although both NLR and IL-6 are efficient biomarkers for the prediction of the disease severity and survival of COVID-19 in this study, none of them is significantly associated with the duration of illness.

The most relevant result of this study is the discovery of the association between the duration of illness and serum levels of IL-8 and sIL-2Rα. Patients with high levels of sIL-2Rα required longer time periods to recover than those with low levels. Moreover, patients with critical illness showed higher levels of sIL-2Rα than those with severe illness, suggesting an association of sIL-2Rα with disease severity. In addition, as *elderly are more prone* to severe *COVID*-*19* (22), a positive correlation between sIL-2Rα and age is indicative of association of sIL-2Rα with the severity of disease. These observations are in line with findings from two previous studies which reported that patients with severe COVID-19 show higher levels of sIL-2Rα than moderate or mild cases (20, 23). As a truncated protein cleaved from IL-2Rα expressed on activated T cells, sIL-2Rα is capable to bind IL-2 and seen as a marker of T cell activation. As IL-2/IL-2R pathway plays a key role in proliferation, differentiation, and function of T cells (24), circulating sIL-2Rα is actively involved in the regulation of T cell immune responses and thus suggested to play a role in disease manifestations (25). Regarding COVID-19, it has been suggested that circulating sIL-2Rα contributes to the lymphopenia through inhibiting IL-2 signaling (26). Taken together, our findings substantiate the current view on a relevant biological role of IL-signaling in the evolution of COVID-19.

The role of IL-8 as a biomarker in COVID-19 is under current debate. Although serum levels of IL-8 are reportedly elevated in patients with COVID-19 as compared to healthy subjects (27, 28), conflicting data have been shown concerning the association of IL-8 concentrations with the disease severity of patients (27–30). In the current study, we did not observe an association of IL-8 concentrations with the severity of COVID-19. To our knowledge, the current study demonstrates for the first time an association between serum levels of IL-8 and the duration of illness in patients with severe COVID-19. Similar to sIL-2α shown above, levels of IL-8 are positively associated with the duration of illness. IL-8 is a proinflammatory cytokine produced by blood cells and many types of tissues, and elevated serum levels of IL-8 have been observed in many diseases (31). Known as neutrophil chemotactic factor, IL-8 plays a major role in the recruitment of neutrophils into the site of infection. As neutrophilia, neutrophilic infiltration, and NLR are considered hallmarked of COVID-19 (32, 33), the association of IL-8 with duration of illness may be suggestive of a role of IL-8 signaling in the evolution of COVID-19. A recent study provides evidence that early polymorphonuclear-myeloid-derived suppressor cells (PMN-MDSC) expansion inhibits SARS-CoV-2 specific T-cell responses, and that the frequency of PMN-MDSC at the time of admission is associated with fatal outcome in patients with COVID-19, with a higher frequency of PMN-MDSC in the non-survivor compared with the survivor group (34). In addition, the frequency of PMN-MDSC is positively correlated with plasma levels of IL-8 at the admission time (34). Therefore, one possible mechanism underlying the role of IL-8 in the evolution of COVID-19 could be that it recruits PMN-MDSC which further inhibits the SARS-CoV-2 specific T-cell responses.

The association of the duration of illness of COVID-19 with circulating sIL-2Rα and IL-8 also provides supportive evidence for therapeutics targeting IL-2 and IL-8 singling pathways. Currently, two clinical trials are investigating the potential of anti-IL-8 monoclonal antibodies, BMS-986253 (35) and reparixin (36), in patients with COVID-19. In addition, the efficacy of low/dose IL-2 administration in improving clinical course and oxygenation parameters in patients with SARS-CoV-2 related acute respiratory diseases is under investigation (37). Our study confirmed the relevance of NLR and IL-6 as prominent biomarkers associated with the disease severity in COVID-19. This indicate that our new findings concerning the impact of serum sIL-2Rα and IL-8 may not be limited by the relatively small size of our cohort, in which the group of patients suffering from critical illness group consisted of only 6 patients, and only 3 out of 69 patients died. However, due to the limited hospital bed capacity during the period from Jan. to Feb. 2020, time from onset to the admission varies substantially among patients. This variation has affected the duration of illness and the time of the laboratory tests, and thus may interfere with accuracy of the analysis. In addition, due to the small size of the cohort, we did not split the data into training and validation set for the examination of association between immunological parameters and clinical outcomes. Therefore, further studies with a larger sample size need to be carried out in order to validate the finding.

Irrespective to these limitations, our study shows that circulating IL-8 and sIL-2Rα are associated with the duration of illness in patients with severe COVID-19. Since IL-8 and sIL-2Rα are essentially involved in the regulations of neutrophils and T cells, respectively, our results shed some new light on the relevance of these cells in evolution of COVID-19 and on mechanisms how these cells are regulated in the disease.

## Data Availability Statement

The raw data supporting the conclusions of this article will be made available by the authors, without undue reservation.

## Ethics Statement

The studies involving human participants were reviewed and approved by Institutional Review Board of Tongji Hospital, Huazhong University of Science and Technology. Written informed consent for participation was not required for this study in accordance with the national legislation and the institutional requirements.

## Author Contributions

XHY, ZXW and APM conceived and supervised the study, XKY, JC, JY, LJZ, CHW, APM collected data, LZ analyzed the data, XHY, FP, LZ and APM wrote the manuscript. All authors contributed to the article and approved the submitted version.

## Funding

This work was supported by the National Natural Science Foundation of China (81600048), Xiamen Science and Technology Bureau (project number: 3502Z20194004), the Deutsche Forschungsgemeinschaft (DFG-27260646 and GRK1727 “Modulation of Autoimmunity”), and the German Center for Lung Research (DZL).

## Conflict of Interest

The authors declare that the research was conducted in the absence of any commercial or financial relationships that could be construed as a potential conflict of interest.
