# Supplementary material for: High Levels of Circulating IL-8 and Soluble IL-2R Are Associated With Prolonged Illness in Patients With Severe COVID-19
Source: Front Immunol. 2021 Jan 29;12:626235. doi: 10.3389/fimmu.2021.626235 (PMC7878368; doi:10.3389/fimmu.2021.626235)
Supplement: Supplementary file 1 [file DataSheet_1.pdf]

Supplementary information

Supplementary table 1. Diagnostic markers for distinguishing COVID-19 patients with severe and critical severe illness

Parameter	Threshold	Sensitivity	Specificity	AUC（95% CI）
**WBC** (x10^9/ml)	13.75	0.667	1.000	0.807（0.496-1.000）
**Neutrophils** (x10^9/ml)	5.79	0.833	0.873	0.831（0.575-1.000）
**NLR**	4.94	0.833	0.810	0.806（0.551-1.000）
**sIL-2Rα** (U/mL)	957.00	0.750	0.960	0.830（0.557-1.000）
**IL-6** (pg/mL)	24.92	1.00	0.961	0.990（0.967-1.000）
**IL-10** (pg/mL)	6.85	0.750	0.940	0.837（0.568-1.000）

The optimal cut-off values (threshold) of the diagnostic markers were calculated by applying the receiver operating curve (ROC) analysis. Area under curve (AUC) was calculated by using the 'auc' function in R software. WBC, white blood cells. NLR, neutrophil-to-lymphocyte ratio.

Supplementary table 2. Correlation between quantitative traits in COVID-19 patients.

	IL-10	NLR	WBC	NE	Age	IL-6	sIL-2Rα	TNF-α	RBC	LY	PLT	IL-8	Duration of illness
**IL-10**	1												
**NLR**	0.47<br>**	1											
**WBC**	0.4<br>*	0.62<br>***	1										
**NE**	0.44<br>**	0.77<br>***	0.96<br>***	1									
**Age**	0.25<br>Ns	0.16<br>Ns	0.05<br>Ns	0.09<br>Ns	1								
**IL-6**	0.25<br>Ns	0.25<br>Ns	0.47<br>**	0.44<br>**	0.33<br>+	1							
**sIL-2Rα**	0.36<br>*	0.41<br>**	0.44<br>**	0.45<br>**	0.52<br>***	0.47<br>**	1						
**TNF-α**	0.31<br>+	0.22<br>Ns	0.43<br>**	0.34<br>*	0.33<br>+	0.58<br>***	0.68<br>***	1					
**RBC**	-0.14<br>Ns	0.19<br>Ns	0.09<br>Ns	0.14<br>Ns	-0.28<br>+	-0.13<br>Ns	-0.21<br>Ns	-0.18<br>Ns	1				
**LY**	-0.11<br>Ns	-0.49<br>***	0.19<br>Ns	-0.08<br>Ns	-0.17<br>Ns	0.11<br>Ns	0.03<br>Ns	0.34<br>*	-0.17<br>Ns	1			
**PLT**	-0.18<br>Ns	-0.2<br>Ns	0.03<br>Ns	-0.03<br>Ns	-0.01<br>Ns	-0.3<br>+	-0.03<br>Ns	-0.14<br>Ns	-0.05<br>Ns	0.22<br>Ns	1		
**IL-8**	0.01<br>Ns	0.04<br>Ns	0.15<br>Ns	0.15<br>Ns	0.15<br>Ns	0.17<br>Ns	0.21<br>Ns	0.01<br>Ns	-0.25<br>Ns	-0.03<br>Ns	0<br>Ns	1	
**Duration of illness**	-0.18<br>Ns	-0.09<br>Ns	-0.17<br>Ns	-0.16<br>Ns	0.21<br>Ns	-0.05<br>Ns	0.27<br>+	0<br>Ns	-0.22<br>Ns	-0.05<br>Ns	0.07<br>Ns	0.13<br>Ns	1

Correlations between variables were analyzed by the ‘rcorr’ function in R, and P values and correlation coefficient were calculated, and *p* values were adjusted for multiple comparison. $^{+}$ p<0.05 but $p^{adjusted}$ >0.05, * $p^{adjusted}$<0.05, ** $p^{adjusted}$ <0.01, *** $p^{adjusted}$ <0.001. NLR, neutrophil-to-lymphocyte ratio. WBC, White Blood Cell. NE, Neutrophil count. LY, lymphocytes. RBC, rec blood cells. PLT, Platelets,.

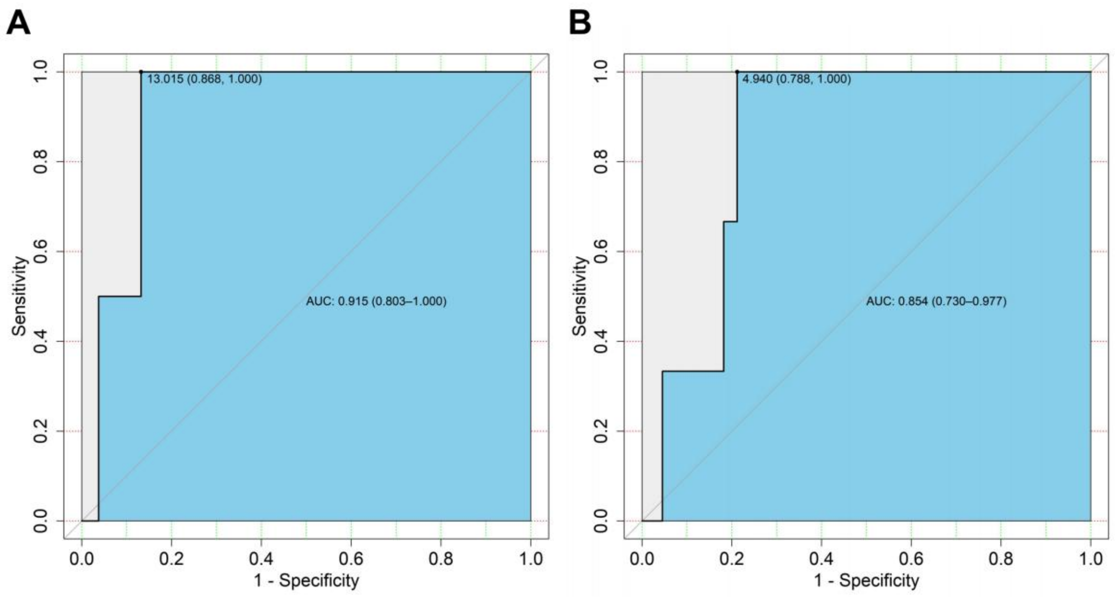

Supplementary figure 1. ROC curves with optimal cut-off points of IL-6 (A) and NLR (B) for the prediction of survival in patients with COVID-19. The optimal cut-off values were determined by minimizing the Manhattan distance on the ROC curve to the left top edge of the diagram where the sum of sensitivity and specificity was maximized. The area under curve (AUC) was calculated by using the 'auc' function in R. Values of optimal cutoff, specificity, sensitivity and AUC are indicated.

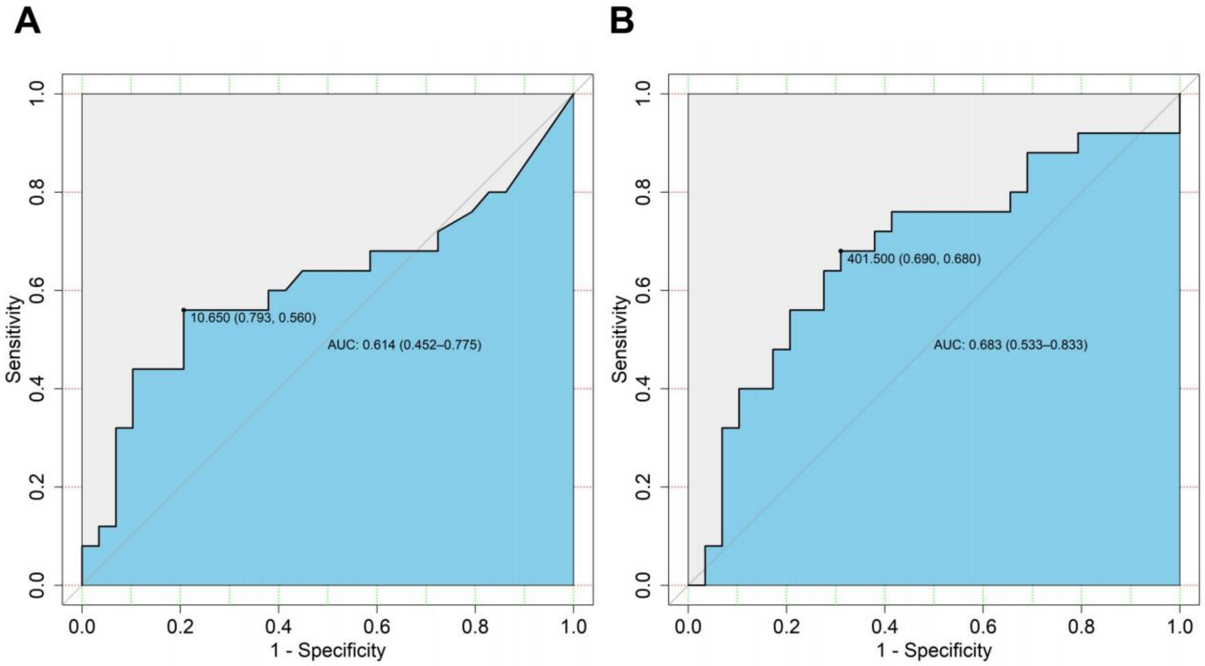

Supplementary figure 2. Receiver operating curve (ROC) curves with optimal cut-off points of IL-8 (A) and sIL-2Rα (B) for the prediction duration of illness in patients with COVID-19. The optimal cut-off values were determined by minimizing the Manhattan distance on the ROC curve to the left top edge of the diagram where the sum of sensitivity and specificity was maximized. The area under curve (AUC) was calculated by using the 'auc' function in R. Values of optimal cutoff, specificity, sensitivity and AUC are indicated.
